# Meeting physical activity guidelines is associated with reduced risk for cardiovascular disease in black South African women; a 5.5-year follow-up study

**DOI:** 10.1186/1471-2458-14-498

**Published:** 2014-05-23

**Authors:** Kasha Dickie, Lisa K Micklesfield, Sarah Chantler, Estelle V Lambert, Julia H Goedecke

**Affiliations:** 1 UCT/MRC Research Unit for Exercise Science and Sports Medicine, Department of Human Biology, University of Cape Town, Cape Town, South Africa; 2 Non-Communicable Disease Research Unit, South African Medical Research Council, PO Box 19070, Parow, Tygerberg 7505, South Africa; 3 MRC/Wits Developmental Pathways for Health Research Unit, Department of Paediatrics, Faculty of Health Sciences, University of Witwatersrand, Johannesburg, South Africa

**Keywords:** Meeting international physical activity guidelines, body composition, cardio-metabolic outcomes

## Abstract

**Background:**

Low levels of physical activity (PA) have been associated with increased risk for cardiovascular disease (CVD) and type 2 diabetes (T2D), but few studies have examined whether meeting international PA guidelines is associated with reduced risk in a black South African (SA) population. The aims of this study were to compare body composition and cardio-metabolic risk factors for CVD and T2D between active and inactive groups (part 1, cross-sectional analysis) and, to determine whether PA level predicts changes in body composition and cardio-metabolic risk factors for CVD and T2D at follow-up after 5.5-years (part 2, longitudinal analysis).

**Methods:**

Part 1 included a sample of 240 apparently healthy black SA women (26 ± 7 years) who underwent the following measurements at baseline: PA (Global Physical Activity Questionnaire (GPAQ)), body composition and regional fat distribution (dual-energy x-ray absorptiometry and computerised tomography), blood pressure, fasting glucose, insulin and lipid concentrations. For part 2, a sub-sample of women (*n* = 57) underwent the same measurements after a 5.5-year period.

**Results:**

At baseline, 61% of women were classified as meeting the guidelines for moderate- to vigorous-intensity physical activity (MVPA) according to GPAQ. Women who were active had significantly lower body weight (p < 0.001), body fat (BMI, fat mass, % body fat, waist circumference, central and appendicular fat mass, all p < 0.001), and measures of insulin resistance (fasting serum insulin and HOMA-IR, both p = 0.01), and higher high-density lipoprotein cholesterol (p = 0.041), compared to the inactive group. At follow-up, all body fat measures increased significantly in both groups and diastolic blood pressure decreased significantly in those who were active at baseline, but did not change in those who were inactive.

**Conclusions:**

Meeting PA guidelines was associated with decreased risk for CVD and T2D in black SA women, but did not prevent the increase in body fat over time. Interventions promoting physical activity to specifically address obesity in this high-risk group are recommended.

## Background

Data from longitudinal cohort studies conducted in the USA have shown that when physical activity (PA) is promoted, it has a positive impact by reducing the risk of cardiovascular disease (CVD) [1,2]. Subsequently, the World Health Organization (WHO) has implemented several public health recommendations to increase PA, in an attempt to reduce disease risk. Accordingly, those who meet the global recommendations of PA for health are referred to as ‘sufficiently active’ and, engage in at least 150 minutes of moderate-intensity activity per week; or 75 minutes of vigorous-intensity activity per week; or an equivalent combination of moderate- to vigorous-intensity physical activity (MVPA) [3].

To date, a wide range of methods has been used to measure PA in adults. These include self-report methods such as questionnaires, activity logs and diaries [4]. The WHO STEPwise approach to chronic disease risk factor surveillance (STEPS) was initiated in 2000, and uses the Global Physical Activity Questionnaire (GPAQ) to collect PA data in both high-income countries (HICs) [5] and low- and middle-income countries (LMICs) [6] defined by the World Bank. Benefits of its use include domain-specific PA data collected during work, transport and leisure time. Results from the most recent South African Demographic Health Survey (SADHS) [7] show that only 14% of women were sufficiently active, the majority of PA was achieved during leisure-time (53.9%), with a 13.1% difference shown between black African urban (49.8%) and rural (62.9%) groups. The contribution of work- and transport-related PA to overall PA (18.4% and 27.6%, respectively) were lower than leisure-time PA, with smaller differences between black African urban and rural groups (5.1% and 8.1%, respectively). In addition, women with no formal education were the most inactive (68%). In contrast, smaller studies in SA have shown black rural women accumulate more activity than black women living in urban settings [8-10]. These results therefore suggest that black urban SA women are a particularly vulnerable group for low levels of habitual PA.

Although previous SA studies have examined the association between PA, body composition and body fat distribution measures [11,12], most have used body mass index (BMI) and waist circumference (WC). To our knowledge there are no studies that have used more precise body composition and body fat distribution measures such as dual-energy x-ray absorptiometry (DXA) and computerised tomography (CT). This is important as PA has been shown to differentially alter body composition within the adipose tissue depots, with some studies showing greater relative changes in visceral adipose tissue (VAT) compared to subcutaneous adipose tissue (SAT) in response to exercise interventions [13,14]. Black SA women have significantly less VAT and more abdominal SAT than white women, despite being more insulin resistant [15-17], however no studies have examined the association between PA and these fat depots in this population.

Cross-sectional data from studies in Sub-Saharan Africa (SSA) [10,18] support evidence from larger international prospective female cohort studies [19,20] showing an inverse association between MVPA and cardio-metabolic disease risk. Results from small-scale SA and Cameroonian adult women studies showed similar inverse associations between PA energy expenditure (PAEE) and fasting plasma glucose levels [10,21], and 2-hour plasma glucose levels [18]. However, it remains unknown if being physically active (meeting global PA recommendations for health) [3] will reduce the risk and onset of CVD and type 2 diabetes (T2D) over time, particularly among women living in LMICs such as SA. Therefore, the aims of this study were i) to compare body composition measures and cardio-metabolic risk factors for CVD and T2D between active and inactive groups and ii) to determine whether baseline PA level predicts changes in body composition and cardio-metabolic risk factors for CVD and T2D at the end of a 5.5-year follow-up period.

## Methods

### Participants

Participants included 240 apparently healthy premenopausal black SA women who were tested in 2005/6, the details of which have been published previously [22,23]. The women were recruited from church groups, community centers, and universities and through the local press, and were included in the study if they were i) 18–45 years old; ii) had no known diseases and were not taking medications for T2D, hypertension, HIV/AIDS, or any other cardio-metabolic diseases; iii) were not pregnant, lactating or postmenopausal (self-reported); and iv) of SA ancestry (self-reported). For the purposes of this study, 9 women were excluded from the original cohort based on invalid PA data in 2005/6 as instructed by the GPAQ Analysis Guide [24].

The original cohort (*n* = 231) of women were contacted and invited to participate in the follow-up study in 2010/11. Of the original sample 126 were non-contactable, 38 women refused to participate in the present study, 8 women disclosed they were HIV-positive and/or taking anti-retroviral medication, 1 woman was deceased, and another woman had recently given birth and was lactating. Voluntary HIV screening was performed and participants were further excluded on the basis of a confirmed positive test (Sanitests Home Test Kits, SA). Those who declined HIV screening were not excluded from the sample. Only 57 women of the original baseline sample underwent follow-up testing and were included in the follow-up subsample analysis. Testing procedures were identical to those at baseline. The same Xhosa-speaking fieldworker involved in the original baseline study assisted with the participant recruitment and testing at follow-up. Ethical approval for this research study (Ref. No. 101/2004) was obtained from the Research and Ethics Committee of the Faculty of Health Sciences, from the University of Cape Town.

### Socio-economic status and behaviour/lifestyle factors

At baseline and follow-up, a socio-demographic questionnaire was administered that included measures of socio-economic status (SES), family history of CVD and T2D (first degree relatives), and behavioural/lifestyle factors. Four indicators of SES were used: education (completion of secondary school), employment, housing density and asset index. An asset index score was based on 14 items reflecting individual and household wealth and resources. This included electricity in the home, ownership of a television, radio, motor vehicle, fridge, stove/oven, washing machine, telephone, video machine, microwave, computer, cellular telephone and paid television channels. Participants were categorised as employed or unemployed. Students were categorised as unemployed. Housing density was defined as the number of persons in the household divided by the number of rooms. Behavioural/lifestyle factors included current smoking status (categorised as smoker or non-smoker), alcohol consumption based on average weekly intake of alcohol (categorised as women who consumed ≥1 drink per day as consumers of alcohol), and hormonal contraceptive use (oral or injectable vs. none).

### Body composition assessment

Weight and height, in lightweight clothing without shoes, were measured using a standard scale and stadiometer, respectively (Detecto, Model UWE BW-150, Cardinal Scale Manufacturers, Webb City, Missouri, USA). WC at the level of umbilicus, and hip circumference at the greatest protuberance of the buttocks were measured.

Whole body composition, including fat mass (FM) and fat-free soft tissue mass (FFSTM), was measured by dual energy x-ray absorptiometry (DXA, Discovery-W®, software version 12.7.3.7; Hologic, Bedford, MA) according to standard procedures. In vivo precision (CV) was 0.7% and 1.67% for FFSTM and FM, respectively. Regional body fat distribution was characterised as central fat mass (CFM) and appendicular fat mass (AFM), as previously described [25]. Those participants whose body proportions exceeded the DXA scanning area were analysed using the arm-replacement method, which replaces the data obtained for the left arm with the data obtained for the right arm [26]. A single-slice computerised tomography (CT) (Toshiba X-press Helical Scanner®; Toshiba Medical Systems, Tokyo, Japan) scan was taken at the level of the L4-L5 lumbar vertebrae to determine VAT and SAT areas [27].

### Cardio-metabolic risk factors for CVD and T2D

Cardio-metabolic measurements including resting blood pressure (BP), fasting serum lipid levels, fasting plasma glucose and serum insulin concentrations were measured at baseline and follow up. After at least 5 minutes of seated rest, BP was measured three times at 1-minute intervals using an appropriate-sized cuff and an automated BP monitor (Omron® 711; Omron Health Care, Hamburg, Germany). An average of the last two readings was used for analyses.

Serum total cholesterol (TC) (intra-assay %CV: 0.4%), triglyceride (TG) (intra-assay %CV: 0.6%), and high-density lipoprotein cholesterol (HDL-C) (intra-assay %CV: 0.55%) concentrations were measured on the Roche Modular Auto Analyzer (Roche/Hitachi Cobas C System from Roche Diagnostics GmbH, D-68298, Mannheim) using enzymatic colorimetric assays. Low-density lipoprotein cholesterol (LDL-C) was calculated using the Friedewald equation [28]. Fasting plasma glucose levels at baseline and follow-up were measured using the glucose oxidase method [(Baseline: Glucose Analyzer 2, Beckman Instruments, Fullerton, CA, USA with intra-assay CV: 0.67%)] [Follow-up: YSI 2300 STAT PLUS, YSI Life Sciences, Yellow Springs, OH with intra-assay CV: 1.17% and inter-assay CV: 2.29%)]. An inter-method comparison was performed and yielded an inter-assay CV of 1.53%. Serum insulin levels were measured by a Micro-particle Enzyme Immunoassay (MEIA) (AxSym Insulin Kit, Abbot, IL, USA) [Baseline: intra-assay CV: 2.3% and inter-assay CV: 3.2%)] [Follow-up: intra-assay CV: 3.28% and inter-assay CV: 5.45%)]. The homeostasis model of insulin resistance (HOMA-IR) was calculated from the fasting insulin and glucose concentrations at both baseline and follow-up [29].

### Physical activity

Physical activity data was collected using the GPAQ [24,30], which has been shown to be similarly reliable and valid for use in SA [7], and with accelerometry as an objective PA measure in a LMIC context [31]. An introductory text explaining which activities to consider for each of the different domains, as well as the definitions of moderate-intensity and vigorous-intensity PA, was read out to each participant. Total PA time recorded in minutes per week (min/week) according to intensity bands (total moderate, total vigorous and MVPA) were calculated for each of the three domains (work, active commuting and leisure).

The WHO STEPwise approach to chronic disease risk factor surveillance (STEPS) uses the GPAQ and GPAQ Analysis Guide criteria to categorise active and inactive groups [24]. The large baseline cohort (*n* = 231) was categorised into the two activity groups. Those who met the following criteria were defined as active: 30 minutes of moderate-intensity activity or walking per day, on at least 5 days in a typical week; or 20 minutes of vigorous-intensity activity per day on at least 3 days in a typical week; or 5 days of any combination of walking and moderate- or vigorous-intensity activities achieving a minimum of at least 600 MET-minutes per week. Those who did not meet these criteria were classified as inactive.

### Statistical analysis

Data are presented as means ± standard deviations, or percentages (*n*). Chi-squared tests were used to examine differences between categorical data (behaviour/lifestyle factors) at baseline and follow-up. Non-normally distributed variables (PA, HDL-C, TG and serum insulin levels) were normalized by log transformation for parametric analyses.

Two-way analysis of covariance (ANCOVA) adjusting for differences in age was used to compare body composition and body fat distribution measures between active and inactive groups. Furthermore, an ANCOVA, adjusting for age, as well as age and weight, was used to compare cardio-metabolic outcomes measured between the two activity groups.

Changes in body composition, body fat distribution and cardio-metabolic outcome measurements over the 5.5-year follow-up period were assessed using repeated measures ANOVA, adjusting for age (for body composition outcomes), and age and weight (for cardio-metabolic outcomes), with Tukey post-hoc analyses. Statistical significance was based on a p < 0.05. All data were analysed using STATISTICA version 10 (StatSoft Inc. Tulsa, OK, USA).

## Results

### Physical activity

**Figure 1:**
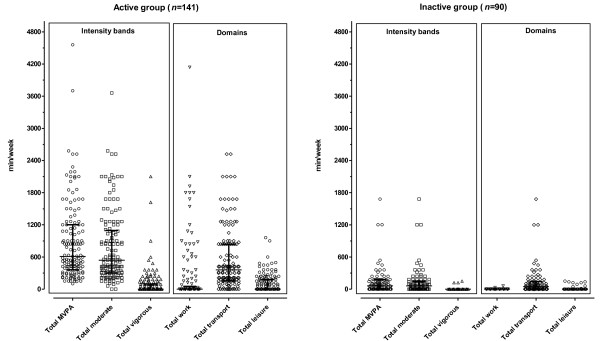
**A diagrammatic comparison of weekly physical activity time between active and inactive groups (medians with inter-quartile ranges presented in Table**1**).**

According to the GPAQ [24] Analysis Guide criteria (http://www.who.int/chp/steps/resources/GPAQ_Analysis_Guide.pdf), 61% of the women were classified as active. The individual and group PA data (min/week) for the active and inactive groups, broken down into the various intensity bands (total moderate, total vigorous and total moderate-vigorous) and domains (work-, transport- and leisure-time) according to the GPAQ criteria, are presented in Figure 1 and Table 1. By design, women classified as active by definition reported more min/week, in all PA domains. Notably, the majority of PA time recorded by both groups was reported for transport (domain) and performed at a moderate intensity (walking).

**Table 1:** Comparison of physical activity time measured using the GPAQ between active and inactive groups

**Physical activity time**	**Active ( *n = *** **141)**	**Inactive ( *n = *** **90)**	**p values**
Total vigorous (min/week)	0 (0–90)	0 (0–0)	<0.001
Total moderate (min/week)	540 (300–1080)	60 (0–140)	<0.001
Total MVPA (min/week)	610 (360–1200)	60 (0–180)	<0.001
***Work***			
Total work (min/week)	0 (0–30)	0 (0–0)	<0.001
***Transport***			
Total travel (min/week)	360 (160–840)	45 (0–140)	<0.001
***Leisure***			
Total leisure (min/week)	0 (0–180)	0 (0–0)	<0.001

Values are medians with inter-quartile range in parenthesis. MVPA, moderate- to vigorous-intensity physical activity. PA time (work, transport and leisure) were normalized by log transformation for parametric analyses (ANOVA).

### Baseline socio-demographic, body composition and body fat distribution characteristics in the PA groups

There were no differences in level of education, housing density or asset index between activity groups, however a larger proportion of women from the inactive group were employed (36.3 vs. 20.3%; p = 0.008). Few women smoked (12.2%) and there were no differences in alcohol consumption or hormonal contraceptive use between the activity groups.

Age at baseline, body composition and body fat distribution measurements of the PA groups are presented in Table 2. The active group was significantly younger than the inactive group (p = 0.019) and therefore all subsequent analyses were adjusted for age. Height was not different between the activity groups, however all other measures of whole body fat were significantly lower in the active compared to the inactive group, even after adjusting for age. All absolute measures of regional body fat distribution, including waist and hip circumferences, central fat mass and appendicular fat mass were significantly lower in the active compared to the inactive group, however when expressed as a % of total fat mass, these differences were no longer significant. In addition, SAT was significantly lower in the active compared to the inactive group; however there was no significant difference in VAT or VAT/SAT.

**Table 2:** **Differences in baseline age**, **body composition and body fat distribution characteristics between the physical activity groups**

	***n***	**Active**	***n***	**Inactive**	**p values**
**Age (years)**	*141*	26 ± 7	*90*	28 ± 8	***0.019***
***Body composition***					**Age-adjusted p values**
Height (m)	*141*	1.60 ± 0.1	*90*	1.60 ± 0.1	*0.463*
Weight (kg)	*141*	71.7 ± 19.1	*90*	83.9 ± 20.6	< ***0.001***
BMI (kg/m^2^)	*141*	28.0 ± 7.5	*90*	32.7 ± 8.0	< ***0.001***
Fat-free soft tissue mass (kg)	*126*	40.9 ± 5.9	*74*	43.1 ± 6.6	***0.025***
Fat-free soft tissue mass (%)	*126*	61.0 ± 7.9	*74*	56.3 ± 6.9	< ***0.001***
Fat mass (kg)	*126*	26.0 ± 12.5	*74*	33.2 ± 12.6	< ***0.001***
Body fat (%)	*126*	35.8 ± 8.5	*74*	40.8 ± 7.3	< ***0.001***
***Body fat distribution***					
Waist circumference (cm)	*141*	86.2 ± 17.0	*90*	95.3 ± 18.2	***0.001***
Hip circumference (cm)	*141*	109.0 ± 15.4	*90*	117.4 ± 14.8	< ***0.001***
Waist:Hip	*141*	0.79 ± 0.1	*90*	0.80 ± 0.1	*0.296*
CFM (kg)	*124*	11.5 ± 6.4	*74*	14.9 ± 6.5	***0.001***
CFM (% FM)	*124*	42.0 ± 7.8	*74*	43.9 ± 4.6	*0.091*
AFM (kg)	*124*	14.5 ± 6.3	*74*	18.3 ± 6.4	< ***0.001***
AFM (% FM)	*124*	56.4 ± 8.8	*74*	55.9 ± 4.6	*0.855*
VAT (cm^2^)	*84*	50.0 ± 39.4	*53*	65.8 ± 36.5	*0.088*
SAT (cm^2^)	*84*	331.6 ± 204.5	*53*	429.8 ± 188.5	***0.044***
VAT/SAT	*84*	0.15 ± 0.1	*53*	0.16 ± 0.1	*0.700*

Values are unadjusted mean ± standard deviation or percentage (*n*). BMI, body mass index; FM, fat mass; CFM, central fat mass; AFM, appendicular fat mass; VAT, visceral adipose tissue; SAT, subcutaneous adipose tissue.

### Baseline cardio-metabolic outcomes in the PA groups

Baseline cardio-metabolic characteristics of the two PA groups are presented in Table 3. The active group had significantly higher HDL-C concentrations, lower fasting serum insulin concentrations and lower HOMA-IR values compared to their inactive counterparts, before and after adjusting for age. However, these differences were no longer significant after adjusting for age and weight. None of the other cardio-metabolic outcomes were significantly different between activity groups.

**Table 3:** Differences in baseline cardio-metabolic outcomes between the physical activity groups

	***n***	**Active**	***n***	**Inactive**	**Optimal range**	**p values**	
***Resting blood pressure***						**Age-adjusted**	**Age-& weight-adjusted**
SBP (mmHg)	*141*	109.6 ± 16.6	*90*	111.3 ± 14.0	100 - 140	*0.682*	*0.389*
DBP (mmHg)	*141*	74.0 ± 11.9	*90*	76.0 ± 9.9	70 - 90	*0.797*	*0.275*
***Lipid profile***							
Total cholesterol (mmol/L)	*135*	3.9 ± 0.8	*86*	4.1 ± 0.9	3.1 - 5.2	*0.711*	*0.240*
LDL-C (mmol/L)	*135*	2.2 ± 0.7	*86*	2.3 ± 0.8	1.0 - 3.0	*0.307*	*0.373*
HDL-C (mmol/L)	*135*	1.3 ± 0.4	*86*	1.2 ± 0.4	1.2 - 1.7	***0.041***	*0.409*
Triglycerides (mmol/L)	*135*	0.7 ± 0.3	*86*	0.8 ± 0.4	0.5 - 2.0	*0.229*	*0.925*
TC/HDL-C	*135*	3.5 ± 3.8	*86*	3.7 ± 3.7	-	*0.870*	*0.815*
***Insulin sensitivity***							
Fasting plasma glucose (mmol/L)	*136*	4.5 ± 0.4	*85*	4.4 ± 0.5	3.1 - 5.5	*0.673*	*0.626*
Fasting serum insulin (mU/L)	*138*	10.1 ± 6.8	*89*	12.4 ± 8.7	-	***0.010***	*0.353*
HOMA-IR	*129*	2.1 ± 1.4	*84*	2.6 ± 2.0	-	***0.010***	*0.332*

Values are unadjusted mean ± standard deviation. SBP, systolic blood pressure; DBP, diastolic blood pressure; LDL-C, low-density lipoprotein cholesterol, HDL-C, high-density lipoprotein cholesterol; TC/HDL-C, total cholesterol to high-density lipoprotein cholesterol ratio; HOMA-IR, homeostasis assessment model of insulin resistance [32]. Normal range values highlighted according to the WHO/International Society for Hypertension (ISH) statement for the management of hypertension [33] and Adult Treatment Panel (ATP) III criteria (2001) [34].

### Physical activity: baseline and follow-up

Similar to the original baseline cohort, in the subsample of women (*n* = 57) who were followed up after 5.5-years, 61.4% (*n* = 35) of the women were classified as active according to the GPAQ criteria, and 38.6% (*n* = 22) of the women were classified as inactive. As with the baseline cohort, the majority of PA time was spent in active commuting (transport domain of the GPAQ), particularly walking.

### Changes in SES, body composition and body fat distribution from baseline to follow-up (5.5-years) in the PA groups

SES (education, employment and asset index) increased significantly in both groups between baseline and follow-up (education: active 41.6 vs. 48.6%, p = 0.03 and inactive 36.3 vs. 45.4%, p = 0.02; employment: active 11.8 vs. 51.4%, p = 0.01 and inactive 31.8 vs. 40.9%, p = 0.02; asset index: active 6 vs. 9 household appliances, p = 0.03 and inactive 7 vs. 9 household appliances, p = 0.03). Although housing density remained unchanged in the active group, it increased significantly in those who were inactive (1.1 vs. 1.4 persons/room, p = 0.03). There was a significant increase in smoking and alcohol consumption between baseline and follow-up in the active participants (smoking: 11.4 vs. 17.1%, p = 0.02 and alcohol consumption: 34.3 vs. 57.1%, p = 0.02), while smoking did not change and alcohol consumption decreased in their inactive counterparts (36.4 vs. 27.3%, p = 0.03). At follow-up, significantly less of the women in both groups were using hormonal contraceptives (active: 40.0 vs. 25.7%, p = 0.02 and inactive: 50.0 vs. 27.3%, p = 0.01). Overall, changes in SES and lifestyle were not different between the two activity groups.

Body weight and DXA measures of body fatness and fat-free soft tissue mass increased significantly in both activity groups over the 5.5-year follow-up period (Table 4). Notably, the mean % increase in central fat mass was greater than that of peripheral fat mass (appendicular fat mass) in both groups (active: 28.1 vs. 14.8% and inactive: 25.1 vs. 11.4%). Although SAT increased significantly over time in both groups (p = 0.004), VAT and VAT/SAT did not change significantly in either group over the 5.5-year follow-up period. Changes in body composition and body fat distribution were not different between the activity groups.

**Table 4:** **Changes in body composition and body fat distribution from baseline to follow**-**up** (**5.5**-**years**) **in the PA groups**

	**Active**			**Inactive**					
	***n***	**Baseline**	**Follow**-**up**	***N***	**Baseline**	**Follow-up**			
**Age (years)**	*35*	25 ± 6^a^	31 ± 6^a^	*22*	28 ± 7^b^	34 ± 7^b^			
							**Age-adjusted p values**		
***Body composition***							**Time**	**Group**	**Interaction**
Height (m)	*35*	1.59 ± 0.1	1.59 ± 0.1	*22*	1.60 ± 0.1	1.60 ± 0.1	-	*0.328*	-
Weight (kg)	*35*	82.0 ± 19.6^a^	89.5 ± 19.2^a^	*22*	91.0 ± 15.6^b^	98.3 ± 13.2^b^	< ***0.001***	*0.087*	*0.730*
BMI (kg/m^2^)	*35*	32.4 ± 7.5^a^	35.4 ± 7.6^a^	*22*	35.4 ± 5.9^b^	38.3 ± 6.1^b^	< ***0.001***	*0.181*	*0.732*
FFSTM (kg)	*35*	44.4 ± 6.5^a^	45.4 ± 6.5^a^	*22*	47.3 ± 5.7^b^	48.0 ± 4.6^b^	***0.051***	*0.127*	*0.825*
Fat mass (kg)	*35*	34.2 ± 14.0^a^	38.9 ± 13.9^a^	*22*	39.5 ± 11.0^b^	44.7 ± 10.3^b^	< ***0.001***	*0.135*	*0.490*
Body fat (%)	*35*	40.7 ± 8.6^a^	43.9 ± 6.4^a^	*22*	43.5 ± 6.3^b^	46.7 ± 5.3^b^	***0.001***	*0.166*	*0.823*
***Body fat distribution***									
Waist (cm)	*35*	97.1 ± 17.9^a^	105.1 ± 17.5^a^	*22*	102.4 ± 15.3^b^	113.6 ± 13.3^b^	***0.001***	*0.323*	*0.181*
Hip (cm)	*35*	116.5 ± 16.2	119.4 ± 14.9	*22*	122.3 ± 10.3	123.7 ± 9.3	*0.702*	*0.185*	*0.856*
Waist: Hip	*35*	0.83 ± 0.1^a^	0.87 ± 0.1^a^	*22*	0.83 ± 0.1^b^	0.91 ± 0.1^b^	***0.001***	*0.455*	*0.117*
CFM (kg)	*34*	15.9 ± 7.3^a^	18.7 ± 7.0^a^	*21*	18.9 ± 6.6^b^	22.2 ± 6.3^b^	< ***0.001***	*0.104*	*0.359*
CFM (%FM)	*34*	37.3 ± 11.1^a^	41.6 ± 10.4^a^	*21*	42.3 ± 10.6^b^	47.1 ± 9.5^b^	< ***0.001***	*0.083*	*0.492*
AFM (kg)	*34*	18.2 ± 7.0^a^	20.1 ± 7.2^a^	*21*	20.6 ± 5.0^b^	22.4 ± 5.2^b^	***0.001***	*0.704*	*0.217*
AFM (%FM)	*34*	43.4 ± 9.4^a^	44.9 ± 10.1^a^	*21*	46.7 ± 7.3^b^	47.8 ± 7.6^b^	***0.014***	*0.239*	*0.997*
VAT (cm^2^)	*25*	58.6 ± 36	75.2 ± 41	*14*	80.8 ± 51	89.1 ± 48	*0.563*	*0.316*	*0.452*
SAT (cm^2^)	*25*	418.7 ± 214^a^	472.5 ± 187^a^	*14*	478.0 ± 142^b^	511.7 ± 122^b^	***0.004***	*0.464*	*0.541*
VAT/SAT	*25*	0.14 ± 0.1	0.16 ± 0.1	*14*	0.16 ± 0.1	0.18 ± 0.1	*0.764*	*0.785*	*0.876*

Values are unadjusted mean ± standard deviation or percentage (n). Matching superscript letters represent significant differences (p < 0.05) between age-adjusted baseline and follow-up values for each activity group, based on Tukey post-hoc analyses. BMI, body mass index; FM, fat mass; CFM, central fat mass; AFM, appendicular fat mass; VAT, visceral adipose tissue; SAT, subcutaneous adipose tissue.

### Changes in cardio-metabolic outcomes from baseline to follow-up (5.5-years) in the PA groups

Changes in the cardio-metabolic characteristics of the two activity groups from baseline to follow-up, adjusting for age, and age and body weight, are presented in Table 5. There was a significant group × time interaction for diastolic blood pressure (DBP), such that DBP decreased in the active group, but did not change in the inactive group, after adjusting for age (p = 0.039), and age and weight (p = 0.035). Total cholesterol, LDL-C and TG serum lipid concentrations did not change over the 5.5-year follow-up period in either activity group, but HDL-C increased significantly in the inactive group after adjusting for age, but not age and weight. Fasting plasma glucose levels did not change over the follow-up period in either of the activity groups. However, fasting serum insulin and HOMA-IR were significantly lower in the active vs. inactive group at baseline and follow-up, after adjusting for age. When adjusting for age and weight, the group difference in fasting serum insulin remained significant; however the difference between the activity groups in HOMA-IR was no longer significant.

**Table 5:** Changes in cardio-metabolic outcomes from baseline to follow-up (5.5-years) in the physical activity groups

	**Active**			**Inactive**			**p values**					
	***n***	**Baseline**	**Follow-up**	***n***	**Baseline**	**Follow-up**	**Age-adjusted**			**Age- and weight-adjusted**		
***Resting blood pressure***							**Time**	**Group**	**Interaction**	**Time**	**Group**	**Interaction**
SBP (mmHg)	*35*	112 ± 14	110 ± 17	*22*	113 ± 13	115 ± 19	*0.135*	*0.831*	*0.479*	*0.483*	*0.990*	*0.446*
DBP (mmHg)	*35*	79 ± 8^a^	73 ± 13^a^	*22*	79 ± 7	81 ± 9	*0.081*	*0.276*	***0.039***	*0398*	*0.288*	***0.035***
***Lipid profile***												
TC (mmol/L)	*35*	3.8 ± 0.9	4.1 ± 0.9	*22*	3.6 ± 0.6	3.9 ± 0.9	*0.384*	*0.296*	*0.870*	*0.198*	*0.380*	*0.733*
LDL-C (mmol/L)	*35*	2.3 ± 0.8	2.5 ± 0.9	*22*	2.0 ± 0.5	2.4 ± 0.8	*0.661*	*0.210*	*0.244*	*0.334*	*0.309*	*0.185*
HDL-C (mmol/L)	*35*	1.1 ± 0.3	1.2 ± 0.3	*22*	1.0 ± 0.4^b^	1.2 ± 0.3^b^	***0.004***	*0.918*	*0.165*	*0.067*	*0.965*	*0.194*
TG (mmol/L)	*35*	0.7 ± 0.3	0.9 ± 0.4	*22*	0.7 ± 0.3	0.9 ± 0.3	*0.307*	*0.581*	*0.827*	*0.279*	*0.361*	*0.918*
TC/HDL-C	*35*	3.6 ± 1.4	3.5 ± 1.0	*22*	3.3 ± 0.7	3.3 ± 0.9	*0.441*	*0.910*	*0.110*	*0.919*	*0.670*	*0.152*
***Insulin sensitivity***												
Fasting plasma glucose (mmol/L)	*35*	4.5 ± 0.4	5.2 ± 1.7	*22*	4.6 ± 0.7	5.1 ± 0.8	*0.849*	*0.654*	*0.821*	*0.402*	*0.720*	*0.402*
Fasting serum insulin (mU/L)	*35*	14.0 ± 7.8^a^	12.4 ± 6.0^b^	*22*	17.8 ± 11.0^a^	16.4 ± 10.5^b^	*0.125*	***0.020***	*0.707*	*0.655*	***0.045***	*0.408*
HOMA-IR	*34*	3.1 ± 1.7^a^	2.8 ± 1.5^b^	*21*	3.7 ± 2.7^a^	3.7 ± 2.1^b^	*0.145*	***0.036***	*0.904*	*0.950*	*0.081*	*0.664*

Values are unadjusted mean ± standard deviation. Matching superscript letters represent significant difference (p < 0.05) between age- and weight-adjusted values, based on Tukey post-hoc analyses. SBP, systolic blood pressure; DBP, diastolic blood pressure; TC, total cholesterol; LDL-C, low-density lipoprotein cholesterol, HDL-C, high-density lipoprotein cholesterol; TG, triglyceride; TC/HDL-C, total cholesterol to high-density lipoprotein cholesterol ratio; HOMA-IR, homeostasis assessment model of insulin resistance; Matsuda index, measure of insulin sensitivity [35].

## Discussion

The main findings of this study were that 61% of apparently healthy urban dwelling, black SA women in this cohort were classified as sufficiently active according to current GPAQ criteria, which are based on international physical activity recommendations for health [3]. Compared to those who failed to meet GPAQ criteria, the active women had lower body weight and body fat measures, as well as higher serum HDL-C concentrations, and were more insulin sensitive. These differences in cardio-metabolic variables between groups were mediated by body weight. However, being active at baseline did not attenuate the increase in weight that occurred over a 5.5-year follow-up period, but was associated with a significant decrease in DBP.

The findings of the cross-sectional component of this study have shown that women who were active were lighter and had less overall body fat compared to their less active counterparts. Notably, we found that waist circumference of the active group was significantly lower than that of the inactive group, but this was largely due to their lower SAT than VAT. International intervention studies have shown that a significant increase in PAEE is associated with a loss of fat mass from both the VAT and SAT regions [14,36]. Failure to show a significant difference in VAT between the groups could partly be explained by the smaller sample that had CT measures, but may also relate to the relatively low VAT levels reported in black women from both the USA and SA [15-17]. Goedecke et al. [17] have showed that although both VAT and SAT were associated with reduced insulin sensitivity in black SA women, SAT was more closely related to insulin sensitivity than VAT. Thus, it seems plausible that a significantly lower SAT in black urban women who are sufficiently active may be linked to an improvement in their overall cardio-metabolic profile.

Of particular concern in the longitudinal component of this study was the significant increase in body weight (±7.3 kg) and fat mass (±4.8 kg) over the 5.5-year follow-up period in the free-living women, irrespective of their baseline activity group. Our results show that being active at baseline did not attenuate the increase in weight that occurred over a 5.5-year period in this sample of urban black SA women. Notably, there was a significantly greater increase in central fat mass compared to peripheral fat mass over the follow-up period in both groups. Furthermore, when using the energy balance-related PA guidelines (≥225 min/week) (ASCM’s 2009 Position Stand on PA and Weight Loss) [37] 61.4% of the women in our study were classified as active, however there was still no difference in weight gain between the active and inactive women over the 5.5-year follow-up period. Recent evidence from the SA National Health And Nutrition Examination Survey (SANHANES-1) [38] has highlighted similar age-related trends in weight gain with the largest increases in BMI and WC reported between the ages of 15 and 45 years. When comparing the secular changes in obesity in SA using the data from the SADHS [7] and SANHANES-1 [38], there are significant increases in the prevalence of obesity in women, in particular black women (15–60 years). Evidence from large international prospective cohort studies [39-41] suggest that weight gain is due in part to aging, changes in PA levels, as well as other lifestyle changes, all of which are difficult to characterise [42]. Although results from Luke et al. [43] suggest activity energy expenditure (AEE) plays a limited role in age-related weight gain over a 3-year period, which stems from a more recent meta-analysis comparing objectively measured AEE amongst adult women from HICs and LMICs [44]. To our knowledge, this is the first SA study to examine longitudinal changes in both weight and adiposity level in black urban women. Unfortunately, we were not able to measure the changes in PA over the follow-up period, which may have limited our ability to show an association between activity levels and changes in body composition. Further, GPAQ is reliant on the subjective recall of activity and is not able to accurately assess the intensity of the exercise bout. Future longitudinal studies in this high risk population, whose rate of obesity exceeds 37% (25–34 years) (SANHANES-1) [38], should measure changes in PA in relation to changes in body composition (and cardio-metabolic outcomes) using objective measures of PA that capture not only the intensity of the activity, but also sedentary time.

At baseline, we also showed that women who were active had higher HDL-C levels and were more insulin sensitive (based on fasting insulin and HOMA-IR) compared to their less active counterparts. However, these effects were mediated by differences in body weight. These findings concur with those from other SA studies [10,12] that showed that active black rural women had significantly higher HDL-C and lower TG concentrations than their inactive counterparts [12]. Furthermore, the most active and overweight (BMI ≥25 kg/m^2^) women in the Transition and Health During Urbanisation of South Africa (THUSA) study had significantly lower LDL-C concentration and LDL-C:HDL-C ratio, as well as lower fasting serum insulin concentrations, than the inactive overweight women [12]. Despite the majority of women being overweight or obese, as well as very few participating in leisure-time PA, the cross-sectional data provides support for the current MVPA guidelines and the health-derived benefits (reduction in body fat and improved measures of insulin sensitivity) amongst those who met PA criteria.

Despite an increase in body weight in both activity groups, we found that the higher level of insulin sensitivity in the active compared to the inactive group at baseline was still significant at follow up. This is an important and novel finding, and suggests that despite large increases in body fat, the effect of being previously active has lasting effects on insulin sensitivity. As mentioned, we were not able to track changes in PA over the 5.5-year follow-up period, so it is not known whether these women maintained their activity level over time. Nonetheless, this finding is of particular relevance in this population who has a high prevalence of insulin resistance and T2D [7,38]. In a recent study reporting the prevalence of T2D in a cohort of black urban adults, which is representative of our sample, Peer et al. [45] found that the prevalence of T2D in women was 13.8% (*n* = 707). However, they found no association between PA levels using the GPAQ and prevalence of T2D, but showed a higher prevalence of physical inactivity in women with T2D compared those who were non-diabetic (11.6 vs. 6.1%, p = 0.035). Future studies are required to confirm our findings using objective measures of PA, as well as direct measures of insulin sensitivity and secretion. Overall, our results highlight the importance of meeting daily PA requirements and its relative influence in reducing cardio-metabolic disease risk.

We also found that despite similar increases in body weight between the activity groups, being active at baseline was associated with a significant decrease in diastolic BP, even after adjusting for age and weight. Potential mechanisms for this finding include sympathetic neural stimulation in response to PA, which results in increased blood flow and vasodilation due to nitric oxide release that provides a stimulus for both acute and chronic changes in vascular function [46]. However, both systolic and DBP remained within a normal range after the 5.5-year period in both groups, and may be due to the relatively young age of the participants. Data from the SANHANES-1 [38] highlights the steady increase in the prevalence of hypertension among SA women across the lifespan (15–60 years). Thus, larger, prospective studies are needed to track possible age-related changes in BP and their association with lifestyle and environmental factors known to affect vascular health [47] in this population.

PA at baseline was not associated with follow-up serum lipid concentrations, which remained relatively low in both groups, despite large increases in body weight over the 5.5-year follow-up period. These findings support evidence from international and other SA studies showing that black women have a more favourable lipid profile when compared to other ethnic groups [48-51] which may be explained, in part, by genetic differences [52-54]. For example, a recent gene-association study amongst black and white SA women (Ellman, personal communication) highlighted a number of ‘protective’ single nucleotide polymorphisms (SNPs) within the cholesteryl ester transfer protein (*CETP B1*/*B2*), lipoprotein lipase (*LPL S*/*X*) and proprotein convertase subtilisin/kexin type 9 (*PCSK9 C*/*X*) genes, which were associated with reduced serum lipid concentrations (LDL-C, TG, TC/HDL-C and TG/HDL-C ratios) in black, but not white women. Other behavioural/lifestyle factors not measured in the study, namely dietary intake could also account for the ethnic differences in serum lipid concentrations.

The strengths of the study include the state-of-the-art measures of body fat and its distribution (DXA and CT). To our knowledge, this is the first study to examine PA as a determinant of change in body composition and cardio-metabolic risk for CVD and T2D over time in black SA women who are at a high risk of CVD. However, numerous limitations should be highlighted and include the use of a convenient sample of women resulting in only 25% of the original sample completing all follow-up tests. In addition, women who refused HIV screening at follow-up were not excluded from the study, which may potentially attenuate the change in body composition measurements. Blood samples for the longitudinal study were not analysed at the same time and using the same assay, which could have increased the variability between measures. Although the intra-assay variability was relatively low, we cannot exclude the possibility that the changes in circulating levels could be due to inter-assay variability. The change in PA level over the 5.5-year follow-up period was not measured. Unfortunately, we were only able to measure PA at baseline and did not measure the subjective changes in PA over the 5.5-year period. Future studies should use objective PA measures (accelerometry) to broaden our knowledge of the relationship between PA at different intensities, and changes in body composition and cardio-metabolic outcomes captured over time [55], particularly in black SA women.

## Conclusions

In conclusion, 61% of black apparently healthy women in the cohort were classified as sufficiently active. The majority of PA reported in both activity groups was for travel-purposes (walking), whereas more structured, leisure-time activity was low in this population group. Those who met the PA guidelines had lower body fat, higher serum HDL-C concentrations, and were more insulin sensitive than their inactive counterparts, with the effects of insulin sensitivity still being present at follow-up. However, being active at baseline did not attenuate the increase in body weight that occurred over a 5.5-year follow-up period, but was associated with a significant decrease in DBP. Overall the findings of this study highlight the importance of meeting PA guidelines to reduce cardio-metabolic risk in black SA women. Barriers and determinants of leisure-time PA amongst young black women in SA need to be identified. Furthermore, public health interventions aimed at promoting daily PA to reduce the burden of obesity and its associated morbidities in SA should be designed, implemented and their effectiveness measured.

## Competing interests

This study was funded, in part, by the Sugar Association of South Africa. The study design, interpretation of data or presentation of information was not influenced in anyway by this funding.

## Authors’ contributions

KD was involved in the conception and design of the research study, data cleaning and analysis, as well as in the drafting and writing manuscript and general management of the research team. SC assisted with data collection, and also the cleaning and analysis of some of the data, and in the writing and editing of the manuscript. JHG and LKM were both involved in the conception and design of the research study, assisted and guided the statistical analysis, in the writing and editing of the manuscript. EVL assisted with editing the manuscript. All authors read and approved the final manuscript.

## Pre-publication history

The pre-publication history for this paper can be accessed here:

http://www.biomedcentral.com/1471-2458/14/498/prepub
